# Isolation of Chavibetol and Methyleugenol from Essential Oil of *Pimenta pseudocaryophyllus* by High Performance Liquid Chromatography

**DOI:** 10.3390/molecules23112909

**Published:** 2018-11-08

**Authors:** Edenilson dos Santos Niculau, Leandro do Prado Ribeiro, Thiago Felipe Ansante, João Batista Fernandes, Moacir Rossi Forim, Paulo Cezar Vieira, José Djair Vendramim, Maria Fátima das Graças Fernandes da Silva

**Affiliations:** 1 Departamento de Química, Universidade Federal de São Carlos (DQ/UFSCar)-Rod. Washington Luís, Km 235, São Carlos CEP 13565-905, SP, Brazil; djbf@ufscar.br (J.B.F.); mrforim@gmail.com (M.R.F.); dpcv@ufscar.br (P.C.V.); dmfs@ufscar.br (M.F.d.G.F.d.S.); 2 Curso de Química, Centro de Ciências Integradas, Universidade Federal do Tocantins, Av. Paraguai, s/n—Esquina com Rua Uxiramas, Araguaína CEP 77824-838, TO, Brazil; 3 Centro de Pesquisa para Agricultura Familiar, Empresa de Pesquisa Agropecuária e Extensão Rural de Santa Catarina (CEPAF/EPAGRI)—Rua Ferdinando Ricieri Tusset S/N, São Cristóvão, Chapecó CEP 89801-970, SC, Brazil; leandroribeiro@epagri.sc.gov.br; 4 Departamento de Entomologia e Acarologia, Universidade de São Paulo, Escola Superior de Agricultura “Luiz de Queiroz” (USP/ESALQ)–Av. Pádua Dias, 11—Agronomia, Piracicaba CEP 13418-900, SP, Brazil; tfansante@gmail.com (T.F.A.); jdvendra@usp.br (J.D.V.)

**Keywords:** natural products, essential oil, HPLC isolation, GC/MS, chavibetol, methyleugenol

## Abstract

A high performance liquid chromatography (HPLC) method was developed for the simultaneous isolation, on a semi-preparative scale, of chavibetol and methyleugenol from the crude essential oil of *P. pseudocaryophyllus* leaves. The purity of the isolated compounds and their quantifications were developed using GC/FID. Chavibetol was isolated with high purity (98.7%) and mass recovery (94.6%). The mass recovery (86.4%) and purity (85.3%) of methyleugenol were lower than those of chavibetol. Both compounds were identified on the basis of spectral analysis. The results suggest that the method can provide chavibetol with high purity, mass recovery, and productivity from crude essential, which will be used in bioassays against stored insect pests.

## 1. Introduction

*Pimenta pseudocaryophyllus* (Gomes) L.R. Landrum, popularly known as “cataia” or “craveiro”, is a native Myrtaceae in Brazil with great abundance in the Atlantic Forest and Cerrado biomes [1]. In Brazil, its leaves are commonly used in the treatment of influenza and fatigue, and as a diuretic and flavoring agent. Ethnopharmacological studies indicate that this species is a promising source of several important metabolites [2,3]. Pharmacological and biological activities of different species in the genus have been described, including potent antibacterial action [4,5,6], antifungal [7], anticonceptive, and anti-inflammatory properties [8].

**Figure 1:**
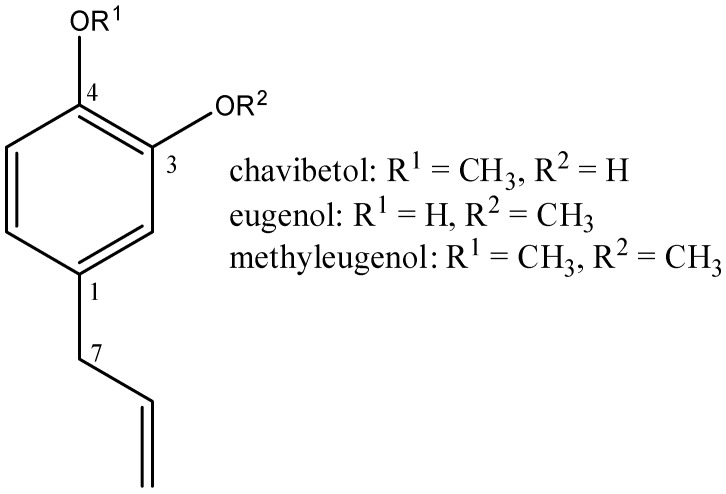
Chemical structures of chavibetol, eugenol, and methyleugenol.

Species of *Pimenta* are sources of the essential oil, which is biosynthesized in the specialized cells of aromatic plants. These species contain secondary metabolites with strong biological activities [9], including insecticidal action (knockdown effect), repellency, feeding, and oviposition deterrence and development inhibition [10]. The essential oil from leaves of *P. pseudocaryophylus* extracted in this study contained phenylpropanoids chavibetol (5-allyl-2-methoxyphenol, Figure 1) and methyleugenol (4-allyl-1,2-dimethoxybenzene, Figure 1) as major components. Chavibetol and other phenylpropanoid compounds are reported as fungicides [11], protecting photosensitization-mediated lipid peroxidation (LPO) of rat liver mitochondria [12] and antioxidants [13].

We previously detected insecticidal activity in the essential oil from the leaves of *P. pseudocaryophylus* against *Sitophilus zeamais* (Coleoptera: Curculionidade), the main insect pest of stored corn [14]. The activity of commercial standard methyleugenol was comparable to that of crude essential oil. However, unfortunately, chavibetol, the main constituent of the oil, is not available commercially. Thus, the purpose of this work was to develop a rapid and sensitive high performance liquid chromatography (HPLC) method for the isolation of chavibetol for further investigation as a potential insecticidal agent. In this regard, while earlier publication described a counter-current chromatography (CCC) method for the isolation of chavibetol from crude essential oil [15], there are no papers published for obtaining of this phenylpropanoid and methyleugenol by HPLC, which is still uncommon. Recently, a semi-preparative reversed-phase HPLC method was reported and shown to be viable and satisfactory for the separation and isolation of carvone from the essential oil of *Mentha spicata* L. [16].

Natural product chemistry using classical methods, for example, open-column chromatography [17], preparative thin-layer chromatography [18], and solvent extraction [17], often produces large amounts of toxic waste, including chlorinated solvents, carcinogens, and environmental contaminants. Methods of isolation developed by HPLC can decrease these undesirable effects, and allow the isolation of large quantities of natural products with high purity in a shorter time; however, this technique is rarely used from crude samples. This approach can be considered a green method, because it can eliminate sample preparation, which is often necessary to isolate chemical compounds, and decreases or eliminate the use of toxic solvents [19,20].

Therefore, HPLC was employed to develop a new method for the isolation of chavibetol and methyleugenol from crude essential oil of *P. pseudocaryophyllus* leaves. This study was also focused on the isolation of chavibetol in high purity, mass recovery, and productivity, which was identified on the basis of spectral analysis such as ^1^H and ^13^C-NMR, and GC/MS. The purity of the isolated compounds and their quantification were developed using GC/FID.

## 2. Results and Discussion

### 2.1. Isolation and Purity of Chavibetol and Methyleugenol

**Figure 2:**
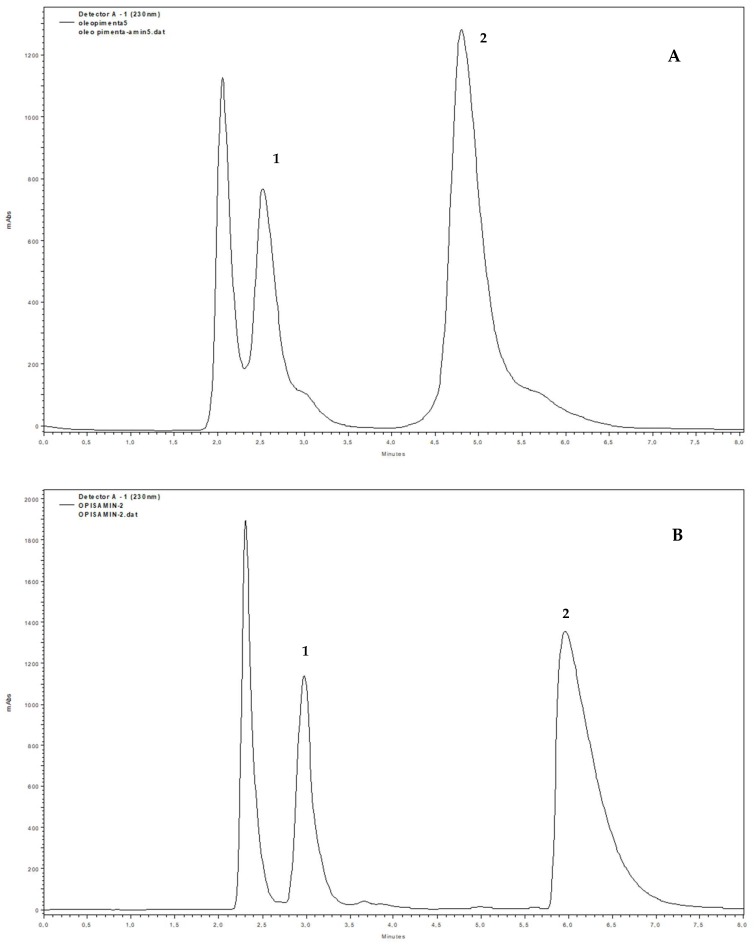
Chromatogram of crude essential oil from the *P. pseudocaryophyllus* leaves obtained from HPLC in analytical (**A**) and semi-preparative scale (**B**). Peak 1 (methyleugenol) and peak 2 (chavibetol).

The HPLC analysis of crude essential oil obtained from *P. pseudocaryophyllus* leaves in analytical mode were optimized with different rates of mobile phase using hexane:ethanol (chromatograms not shown). The best chromatographic resolution was achieved using an isocratic solvent system, hexane:ethanol (92:8) (Figure 2A). Preliminary analysis of the essential oil by ^1^H-NMR showed the presence of eugenol, methyleugenol, and chavibetol, and structural identification of the first two was also supported by comparison with those spectra of commercial standards. The ^1^H-NMR of essential oil also showed higher concentrations (integration area) of chavibetol in comparison with other compounds in this matrix.

Based on the analytical conditions, the scaling of the flow rate to be used in semi-preparative analysis mode was performed, obtaining a flow rate of 6 mL/min using Equation (1), where *S* = scaling factor; *Rα* = diameter of analytical column; *Rp* = diameter of preparative column; *Lα* = length of analytical column; *Lp* = length of preparative column.

(1) $$S=\frac{R_{p}^{\;\;2}\;\cdot L_{p}}{R_{a}^{\;\;2}\;\cdot L_{a}}$$

The semi-preparative scale in the normal elution mode allowed us to isolate 102.7 mg of chavibetol (fraction represented by peak 2 with retention time 6.1 min) and 27.9 mg of methyleugenol (fraction represented by peak 1 with retention time 3.0 min) (Figure 2B) (Table 1) after 11 injections of 130 µL of the crude essential oil from *P. pseudocaryophyllus* leaves at concentration of 143 mg/mL.

**Figure 3:**
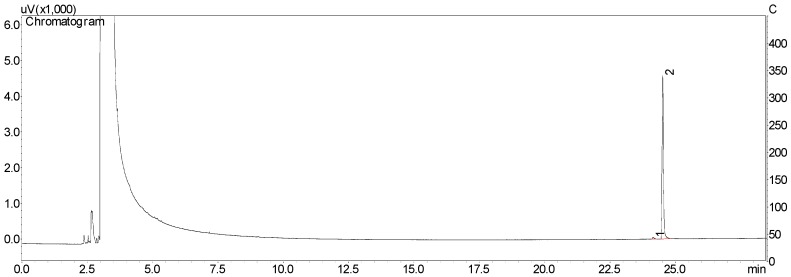
GC/FID chromatogram from the isolated fraction containing chavibetol (peak 2, 98.7%).

**Figure 4:**
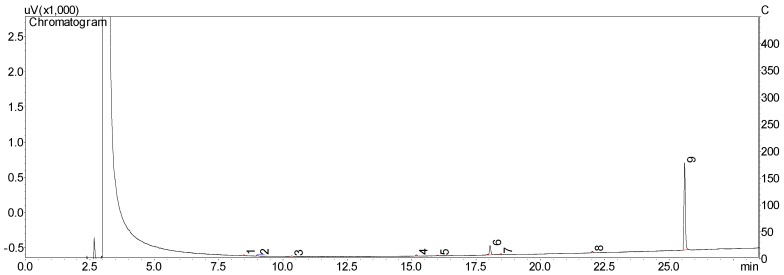
GC/FID chromatogram from the isolated fraction containing methyleugenol (peak 9, 85.3%).

Area percentages (calculated by calibration curve) of chavibetol and methyleugenol in essential oil was 51.7% and 15.4%, respectively. The purity of isolated compounds was determined by GC/FID in triplicate after joint and evaporation of collected fractions. The purity of chavibetol was 98.7% (Figure 3) and of methyleugenol was 85.3% (Figure 4).

As shown in Table 1, the method allows the isolation of chavibetol in high purity and mass recovery (yield), as well as excellent productivity (amount of material collected per unit time) and low solvent consumption per mass isolated, showing that HPLC is an excellent technique to isolate this compound on a semi-preparative scale. Methyleugenol was isolated with lower purity than chavibetol, but with good mass recovery, productivity, and moderate consumption of solvent per isolated mass (Table 1).

### 2.2. Characterization of Chavibetol and Methyleugenol

**Figure 5:**
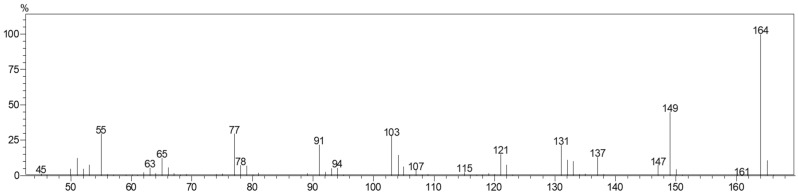
GC/MS mass spectrum of the isolated fraction containing chavibetol. 70 eV electron impact.

The GC/MS analysis of the fraction containing chavibetol showed molecular ion at *m*/*z* 164 (Figure 5). The retention index calculated (1367) is consistent with that reported by dos Santos et al. [15] (1372) and Pino et al. [21] (1374). Analysis by ^1^H-NMR showed the spectral information described in Table 2. These data also are consistent with those previously published by dos Santos et al. [15] and Momin et al. [22].

**Figure 6:**
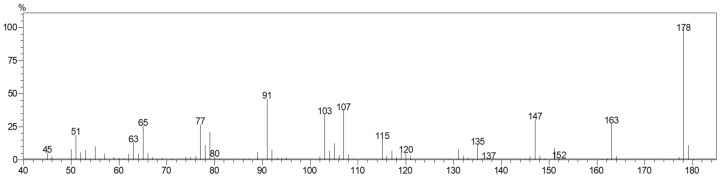
GC/MS mass spectrum of the isolated fraction containing methyleugenol. 70 eV electron impact.

Mass spectrum of fraction containing methyleugenol (Figure 6) and retention time were identical to standard (Sigma-Aldrich, St. Louis, MO, USA). The retention index calculated (1398) is also consistent with that reported by Adams, 2007 (1403). Analyses by ^1^H-NMR described in Table 2 are supported by the data published by Meepagala et al. [23].

## 3. Materials and Methods

### 3.1. Chemicals and Material

Standards eugenol (Figure 1) and methyleugenol were purchased from Sigma-Aldrich (St. Louis, MO, USA). Ethanol, hexane, and ethyl acetate was used in HPLC grade purchased from Panreac (Barcelona, Spain). Deuterated chloroform used in NMR analyses was obtained from Cambridge Isotope Laboratories, Inc. (Andover, MA, USA).

### 3.2. Plant Material and Essential Oils Extraction

The leaves of *P. pseudocaryophyllus* used in this study were collected on 26 June 2011, from samples cultivated in District Caiçara de Pedrinhas, city of Ilha Comprida, São Paulo State, Brazil (24°54′09.2″ S; 47°47′10.8″ W; height: 31 m). A voucher specimen, previously identified by Professor Dr. João Vicente Coffani Nunes (UNESP, Campus Registro) is deposited at ESA herbarium of the Department of Biological Sciences, Luiz de Queiroz College of Agriculture/University of São Paulo (ESALQ/USP) in Piracicaba, São Paulo State, Brazil, under registration No. 121119. After collection, the leaves were separated from other structures and were used in fresh form for essential oil extraction.

Fresh leaves were separated in samples of 100 g each, washed in water, cut into small pieces to optimize extraction by increasing contact surface, and subjected to hydrodistillation using a Clevenger type apparatus for 2 h at 110 °C. The mixture of water and oil (hydrolate) was separated by decantation and dried by adding anhydrous sodium sulfate (Na_2_SO_4_). The extraction yield was 0.81%, determined based on the fresh weight of the plant material (g of oil/g of fresh leaves). The essential oil obtained was stored in a domestic refrigerator (c.a. −10 °C) until analysis.

### 3.3. HPLC Analysis and Isolation

The HPLC analysis and isolation were performed on a HPLC Shimadzu (Kyoto, Japan) composed of an LC-6AD pump, an SPD-10AV VP UV-Vis detector, an SCL-10 VP system controller with an analytical flow cell or a preparative flow cell according to the HPLC mode (analytical or semi-preparative) and a loop of 200 μL.

Analytical conditions were optimized with different proportions of hexane/ethanol (10:90, 70:30 and 92:8) using a Phenomenex Luna amino column (4.6 mm × 150 mm, 10 μm) with flow rate 1 mL/min, 10 µL of injection and detection at 230 nm. Optimal analytical chromatography was carried out under isocratic conditions with the mobile phase constituted of hexane:ethanol (92:8) at flow rate 1.0 mL/min using a Phenomenex Luna amino column (4.6 mm × 150 mm, 10 μm) and detection at 230 nm.

Compound isolations were carried out by the semi-preparative condition on the same HPLC equipment with a Phenomenex Luna amino column (10 mm × 250 mm, 10 μm) with flow rate 6.0 mL/min under the same mobile phase and detection of analytical conditions. For the analytical conditions, crude essential oils were diluted in hexane:ethanol (92:8) at a concentration of 1 mg/mL, and 10 µL was injected. For the semi-preparative conditions, 210 mg of crude essential oil was diluted in hexane:ethanol (92:8) at a concentration of 143 mg/mL and 130 µL was injected. The fraction solvent obtained was evaporated under a vacuum and the purity of isolated compounds was determined by GC/FID and characterized by GC/MS and NMR.

### 3.4. GC/MS Analysis

The qualitative analysis of essential oil constituents was performed using a gas chromatograph 17A (Shimadzu Corporation, Kyoto, Japan) hyphenated to a mass spectrometer (GC/MS) QP5000. The following conditions were used: fused-silica capillary column DB-5MS with 30 m × 0.25 mm ID × 0.25 μm thick film, flow of 1.2 mL/min of helium as carrier gas (99.999%); 1.0 µL of injection volume in ethyl acetate; split ratio of 1:12; injector temperature of 250 °C; detector temperature of 280 °C. Temperature was set at 50 °C for 1.5 min, followed by 4 °C/min to 200 °C, then 10 °C to 250 °C finishing with isotherm of 5 min at 250 °C. Mass spectrum was conducted at 70 eV with a rapid scan of 0.5 scan/s in the mass range 45–500 Da. Volatile components were identified by comparison of their mass spectra with spectra reported in the literature [24], as well with equipment database spectra (NIST11, WILEY8, NIST05, NIST21 and NIST107), comparing their retention indices with those in the literature. Retention indices (RI) were determined using a homologous series of *n*-alkanes (C_9_H_20_–C_20_H_42_) injected under the same chromatographic conditions of the samples using the equation proposed by Van den Dool and Kratz [25].

### 3.5. Purity Analysis of the Isolated Compound and Quantification by GC/FID Analysis

Purities of chavibetol and methyleugenol were performed on GC/FID 17A after injection of 0.1 mg/mL of each compound in triplicate and estimated by normalization of peak areas (%).

Quantification of chavibetol and methyleugenol in the essential oil was carried out by internal standardization as follows: A stock solution of chavibetol (15 mg/mL) and methyleugenol (15 mg/mL) isolated were prepared in ethyl acetate. Each solution was diluted to 2.4, 1.8, 1.2, and 0.6, and 0.15 mg/mL and 1 mg/mL of thymol was added as internal standard. The calibration curve was constructed using linear regression, and the R^2^ coefficients of 0.99 were obtained. A solution of 1.4 mg/mL of the essential oil and 1 mg/mL of thymol in ethyl acetate was injected to calculate the mass percent of chavibetol and methyleugenol in the essential oil by means of ratios between concentrations of isolated compounds in the calibration curve and oil concentration.

Essential oil and isolated compounds were injected using the same chromatographic conditions of GC/MS (Section 3.4), but using a DB-1 column 30 m × 0.25 mm ID × 0.25 μm thick film and using N_2_ as carrier gas.

### 3.6. NMR Analysis

NMR spectra of chavibetol and methyleugenol were performed on a Bruker Avance III spectrometer (Bruker, Billerica, MA, USA) of 9.4 Tesla (400 MHz for the frequency of ^1^H and 100 MHz for ^13^C in Norell tubes of 5 mm id). Chemical shifts (δ) were expressed in parts per million (ppm) using deuterated chloroform (CDCl_3_, 7.3 ppm) and tetramethylsilane (TMS, 0 ppm) as reference standard for ^1^H-NMR and chloroform (77.0 ppm) for ^13^C-NMR.

## 4. Conclusions

The present HPLC method is simple and useful for the simultaneous isolation, on a semi-preparative scale, of chavibetol and methyleugenol from crude essential oil. In addition, no sample preparation is required, small amounts of solvent were used, and the time of analysis was reduced compared to conventional isolation methods such as open column chromatography and preparative thin layer chromatography. Moreover, evaporation of the mobile phase hexane and ethanol was facilitated because they are more volatile than the solvents used in reverse-mode elution. This is the first time that a HPLC method was developed to isolate phenylpropanoids from crude essential oil. Finally, these results show that chavibetol was obtained in high purity, high mass recovery, and high productivity from crude essential oil to be used in bioassay against stored insect pests.

## Figures and Tables

**Table 1:** Summary of the results for the isolation of chavibetol and methyleugenol by HPLC.

Parameter	Chavibetol	Methyleugenol
Isolated mass (mg)	102.7	27.9
Purity (%)	98.7	85.3
Mass recovery (%)	94.6	86.4
Processing time	11 injections: 1.5 h	11 injections: 1.5 h
Solvent consumption (mL)	528	528
Productivity (mg/h)	68.5	18.6
Solvent consumption/isolated mass (mL/mg)	5.1	18.9

**Table 2:** ^1^H (400 MHz, CDCl_3_) and ^13^C-NMR (100 MHz, CDCl_3_) data of methyleugenol and chavibetol.

Position	Methyleugenol		Chavibetol	
	^1^H-NMR	^13^C-NMR	^1^H-NMR	^13^C-NMR
1	-	132.6	-	133.4
2	6.72 (d, 1H, *J* = 2.0 Hz)	111.2	6.79 (d, 1H, *J* = 2.1 Hz)	114.8
3	-	147.4	-	144.9
4	-	148.9	-	145.5
5	6.81 (d, 1H, *J* = 7.9 Hz)	120.4	6.80 (d, 1H, *J* = 8.2 Hz)	119.8
6	6.73 (dd, 1H, *J* = 7.9 and 2.0 Hz)	111.8	6.68 (dd, 1H, *J* = 8.2 and 2.1 Hz)	110.6
7	3.35 (dl, 2H, *J* = 6.9 Hz)	39.8	3.30 (d, 2H, *J* = 6.7 Hz)	39.6
8	5.97 (tdd, 1H, *J* = 12.0, 10.0 and 6.9 Hz)	137.7	5.95 (m, 1H)	137.6
9	5.07 (m, 2H)	115.6	5.07 (m, 2H)	115.5
3-OCH_3_	3.88	55.9	-	-
4-OCH_3_	3.87	55.8	3.88 (s, 3H, –OCH_3_)	56.0
OH	-	-	5.60 (s, 1H, –OH)	-

## References

[1] Landrum L.R., Kawasaki M.L. (1997). The genera of Myrtaceae in Brazil: An illustrated synoptic treatment and identification keys. Brittonia.

[2] Paula J.A.M., Rocha J.B., Nascimento P.M.S., Rezende M.H., Paula J.R. (2006). Estudo farmacognóstico da casca de *Pimenta pseudocaryophyllus* (Gomes) L.R. Landrum-Myrtaceae. Revista Eletrônica de Farmácia.

[3] Paula J.A.M., Paula J.R., Bara M.T.F., Rezende M.H., Ferreira H.D. (2008). Estudo farmacológico das folhas de *Pimenta pseudocaryophyllus* (Gomes) L.R. Landrum-Myrtaceae. Rev. Bras. Farmacogn..

[4] Lima M.E.L., Cordeiro I., Young M.C.M., Sobra M.E.G., Moreno P.R.H. (2006). Antimicrobial activity of the essential oil from two specimens of *Pimenta pseudocaryophyllus* (Gomes) L.R. Landrum (Myrtaceae) native from São Paulo State Brazil. Pharmacologyonline.

[5] Paula J.A.M., Paula J.R., Pimenta F.C., Rezende M.H., Bara M.T.F. (2009). Antimicrobial activity of the crude ethanol extract from *Pimenta pseudocaryophyllus*. Pharm. Biol..

[6] Custódio D.L., Burgo R.P., Moriel B., Barbosa A.M., Rezende M.I., Daniel J.F.S., Pinto J.P., Bianchini E., Faria T.J. (2010). Antimicrobial activity of essential oils from *Pimenta pseudocaryophyllus* and *Tynanthus micranthus*. Braz. Arch. Biol. Technol..

[7] Sanches B.G., Custódio D.L., Faria T.J. Estudo do extrato em acetato de etila de *Pimenta pseudocaryophyllus*. Proceedings of the XVI Encontro Anual de Iniciação Científica.

[8] Paula J.A.M., Silva M.R.R., Costa M.P., Diniz D.G.A., Sá F.A.S., Alves S.F., Costa E.A., Lino R.P., Paula J.R. (2012). Phytochemical analysis and antimicrobial, antinociceptive, and anti-Inflammatory activities of two chemotypes of *Pimenta pseudocaryophyllus* (Myrtaceae). Medicine.

[9] Stefanello M.E.A., Pascoal A.C.R.F., Salvador M.J. (2011). Essential oils from *Neotropical Myrtaceae*: Chemical diversity and biological properties. Chem. Biodiv..

[10] Ebadollahi E. (2013). Essential oils isolated from Myrtaceae family as natural insecticides. Annu. Rev. Res. Biol..

[11] Evans P.H., Bowers W.S., Funk E.J. (1984). Identification of fungicidal and nematocidal components in the leaves of *Piper betle* (Piperaceae). J. Agric. Food Chem..

[12] Mula S., Banerjee D., Patro B.S., Bhattacharya S., Barik A., Bandyopadhyay S.K., Chattopadhyay S. (2008). Inhibitory property of the *Piper betel* phenolics against photosensitization-induced biological damages. Bioorg. Med. Chem..

[13] Rathee J.S., Patro B.S., Mula S., Gamre S., Chattopadhyay S. (2006). Antioxidant activity of *Piper betel* leaf extract and its constituents. J. Agric. Food Chem..

[14] Ribeiro L.P., Ansante T.F., Niculau E.S., Pavarini R., Silva M.F.G.F., Seffrin R.C., Vendramim J.D. (2015). *Pimenta pseudocaryophyllus* Derivatives: Extraction Methods and Bioactivity Against *Sitophilus zeamais* Motschulsky (Coleoptera: Curculionidae). Neotrop. Entomol..

[15] dos Santos B.C.B., da Silva J.C.T., Guerrero P.G., Leitão G.G., Barata L.E.S. (2009). Isolation of chavibetol from essential oil of *Pimenta pseudocaryophyllus* leaf by high-speed counter-current chromatography. J. Chromatogr. A.

[16] Do T.K.T., Hadji-Minaglou F., Antoniotti S., Fernandez X. (2014). Secondary metabolites isolation in natural products chemistry: Comparison of two semipreparative chromatographic techniques (high performance liquid chromatography and high performance thin-layer chromatography). J. Chromatogr. A.

[17] Cornelio V.E., Maluf F.V., Fernandes J.B., da Silva M.F.G.F., Oliva G., Guido R.V.F., Vieira P.C. (2014). Isolation of Tiliroside from *Spiranthera odoratissima* as Inhibitor of *Trypanosoma cruzi* Glyceraldehyde-3-phosphate Dehydrogenase by Using Bioactivity-Guided Fractionation. J. Braz. Chem. Soc..

[18] Rabêlo S.V., Costa E.V., Barison A., Dutra L.M., Nunes X.P., Tomaz J.C., Oliveira G.G., Lopes N.P., Santos M.F.C., Almeida J.R.G.S. (2015). Alkaloids isolated from the leaves of atemoya (*Annona cherimola* × *Annona squamosa*). Rev. Bras. Farmacogn..

[19] Płotka-Wasylka J. (2018). A new tool for the evaluation of the analytical procedure: Green analytical procedure index. Talanta.

[20] Gałuszka A., Migaszewski Z.M., Konieczka P., Namieśnik J. (2012). Analytical Eco-Scale for assessing the greenness of analytical procedures. Trends Anal. Chem..

[21] Pino J.A., Marbot R., Marti M.P. (2006). Chemical composition of the essential oil of *Helenium amarum* (Raf.) H. Rock from Cuba. J. Essent. Oil Res..

[22] Momin R.A., Ramsewak R.S., Nair M.G. (2000). Bioactive compounds and 1,3-Di[(cis)-9-octadecenoyl]-2-[(cis,cis)-9,12-octadecadienoyl]glycerol from *Apium graveolens* L. Seeds. J. Agric. Food Chem..

[23] Meepagala K.M., Sturtz G., Wedge D.E. (2002). Antifungal constituents of the essential oil fraction of *Artemisia dracunculus* L. var. *dracunculus*. J. Agric. Food Chem..

[24] Adams R.P. (2007). Identification of Essential Oil Components by Gas Chromatography/Mass Spectroscopy.

[25] Van Den Dool H., Kratz P.D.J. (1963). A generalization of the retention index system including linear temperature programmed gas—Liquid partition chromatography. J. Chromatogr. A.

